# Treatment variation in patients diagnosed with early stage breast cancer in Alberta from 2002 to 2010: a population-based study

**DOI:** 10.1186/s12913-015-0680-z

**Published:** 2015-01-22

**Authors:** Stacey Fisher, He Gao, Yutaka Yasui, Kelly Dabbs, Marcy Winget

**Affiliations:** School of Public Health, University of Alberta, Edmonton, Alberta Canada; Department of Surgery, University of Alberta, Edmonton, Alberta Canada; Department of Medicine, Stanford University, Palo Alto, CA USA

**Keywords:** Breast cancer, Population-based, Radiotherapy, Surgery

## Abstract

**Background:**

Breast-conserving surgery (BCS) followed by radiotherapy is generally the preferred treatment for women diagnosed with early stage breast cancer. This study aimed to investigate the proportion of patients who receive BCS versus mastectomy and post-BCS radiotherapy, and explore factors associated with receipt of these treatments in Alberta, Canada.

**Methods:**

A retrospective population-based study was conducted that including all patients surgically treated with stage I-III breast cancer diagnosed in Alberta from 2002–2010. Clinical characteristics, treatment information and patient age at diagnosis were collected from the Alberta Cancer Registry. Log binomial multiple regression was used to calculate stage-specific relative risk estimates of receiving BCS and post-BCS radiotherapy.

**Results:**

Of the 14 646 patients included in the study, 44% received BCS, and of those, 88% received post-BCS radiotherapy. The adjusted relative risk of BCS was highest in Calgary and lowest in Central Alberta for all disease stages. Relative to surgeries performed in Calgary, those performed in Central Alberta were significantly less likely to be BCS for stage I (RR = 0.65; 95% 0.57, 0.72), II (RR = 0.58; 95% 0.49, 0.68), and III (RR = 0.62; 95% CI: 0.37, 0.95) disease, respectively, adjusting for patient age at diagnosis, clinical and treatment characteristics. No significant variation of post-BCS radiotherapy was found.

**Conclusions:**

Factors such as region of surgical treatment should not be related to the receipt of standard care within a publicly-funded health care system. Further investigation is needed to understand the significant geographic variation present within the province in order to identify appropriate interventions.

**Electronic supplementary material:**

The online version of this article (doi:10.1186/s12913-015-0680-z) contains supplementary material, which is available to authorized users.

## Background

Standard surgical treatment for breast cancer has evolved over the last three decades leading to significantly improved outcomes [1]. Many women diagnosed with early stage breast cancer have the option of receiving either mastectomy or breast-conserving surgery (BCS) followed by adjuvant radiotherapy. This option is due to results of several clinical trials and meta-analyses which have demonstrated equivalent survival for women who receive these two treatment options [2-4]. BCS with radiotherapy is recommended as the preferred treatment option because BCS is a less invasive procedure, provides a better cosmetic outcome and is associated with less morbidity compared to mastectomy [5]. Research also suggests that those who receive mastectomy have significantly inferior body image, poorer sexual function, lower quality of life and a lower surgical satisfaction rate compared to those who receive BCS [6-9]. Receipt of BCS has been found to be associated with patient age and socioeconomic status in addition to tumor characteristics [10-13].

The receipt of radiotherapy is a vital component of treatment for women who receive breast-conserving surgery. A recent meta-analysis found that the 15-year breast cancer mortality risk was reduced by 5.4% (30.5% versus 35.9%) and the 5-year local recurrence risk was reduced by 19% (7% versus 26%) among women with early stage breast cancer who received BCS with versus without radiotherapy [14]. Receipt of radiotherapy after BCS has been found to be associated with patient age and race, geography, operating surgeon, and patient distance from radiotherapy facilities [15-17].

The purpose of this study was to: 1) assess the proportion of early stage breast cancer patients surgically treated with BCS versus mastectomy, 2) assess the proportion of those treated with BCS who subsequently receive radiotherapy, and 3) explore factors associated with the treatments received in Alberta, Canada. Alberta has a publicly-funded health care system, therefore, assessing variation in care and patient outcomes are important accountability factors for Alberta Health Services, the provincial provider of healthcare. Understanding treatment patterns and variation in jurisdictions is useful for informing potential strengths and issues that may exist in other similar jurisdictions and possibly dissimilar jurisdictions.

## Methods

All cases of stage I, II or III breast cancer (International Classification of Diseases for Oncology [ICD-O-3] code c50 [18]) diagnosed in adult women from 2002 to 2010 who received breast cancer surgery were identified from the Alberta Cancer Registry. Cases of breast cancer were excluded if: 1) the cancer was not the first primary breast cancer diagnosis; 2) if the histology indicated it was a sarcoma, lymphoma or hematopoietic tumor and, 3) if the patient had another cancer diagnosis within 6 months prior to the breast cancer case, for this may influence breast cancer treatment decisions. Patient age, clinical, and treatment information were obtained from the cancer registry including: date of diagnosis; age at diagnosis; estrogen and progesterone receptor (ER/PR) status; cancer stage; type of surgery; geographic region of surgery; receipt of neo-adjuvant and adjuvant chemotherapy; receipt of post-operative radiotherapy; and receipt of hormone therapy. Cancer stage was determined using the American Joint Committee on Cancer (AJCC) 5^th^ edition staging rules for years 2002 and 2003 and the 6th edition for years 2004–2010 [19,20]. Geographic region of surgery was categorized into the five administrative health zones of Alberta. The province of Alberta consists of an area of 662,000 km^2^ and has a population of 3.7 million. Approximately 80% of the population reside in urban areas [21]. Patients diagnosed in 2002 and 2003 who received hormone therapy were classified as ER/PR positive, while those who did not receive hormone therapy were classified as ER/PR negative, since estrogen and progesterone receptor data was not collected in these years. If ER/PR status was missing in patients diagnosed from 2004 to 2010, the case was assumed to be ER/PR positive (N = 108), since roughly 75% of breast cancers in North American are ER/PR positive [22]. Sensitivity analysis found that this assumption did not affect the study results. The Alberta Cancer Registry was awarded the highest level of certification by the North American Association of Central Cancer Registries in all years of the study for its high level of completeness and for the timeliness of data collection and reporting.

Data analyses were performed on two different outcomes: receipt of BCS and receipt of radiotherapy after BCS. First, descriptive statistics were calculated for the demographic, clinical, and treatment characteristics of the breast cancer patients by receipt of BCS and stage at diagnosis; Chi-square and Fisher exact tests were used to determine statistical differences. Log-binomial multiple regression was then used to calculate stage-specific relative risk (probability ratio) estimates of receiving BCS by the following factors: age at diagnosis; geography of surgery; year of diagnosis; ER/PR status; and neo-adjuvant chemotherapy status. Interaction between year of diagnosis and geography of surgery was also assessed through an interaction model; interaction effects were displayed separately from the main effect model stratified by year of diagnosis and geography of surgery to enable easier interpretation (shown in Additional file 1: Tables S1-1, S1-2 and S1-3). The log-binomial regression method provides relative risk estimates as a measure of association using binary outcome data which are easier to interpret than odds ratios obtained from the more traditional logistic regression model.

The same data analysis procedures described above were used to assess associations with receipt of radiotherapy after BCS with slight modifications. For this outcome, hormone therapy was combined with ER/PR status as a single variable to assess whether ER/PR positive patients who did not receive hormone therapy were also less likely to receive radiotherapy after BCS. Secondly, adjuvant chemotherapy status was included in the regression model because receipt of adjuvant chemotherapy could affect whether a patient received post-surgical radiation. Log-binomial regression with the COPY method was used [23].

All statistical analyses were performed using SAS statistical software version 9.3 (SAS Institute, Cary, NC, USA). This study received ethics approval from the Health Research Ethics Board of the University of Alberta.

## Results

There were 14 933 patients diagnosed with stage I, II or III first-ever breast cancer diagnosed in Alberta from 2002 to 2010, excluding patients with non-solid tumors and those who had another cancer diagnosis within 6 months prior to their breast cancer diagnosis. Patients who were not treated with surgery (281 patients) and those who received an unknown or other surgery type were also excluded (6 patients). The final cohort consists of 14 646 patients.

Patient age, clinical and treatment characteristics and their associations with receipt of BCS are shown in Table 1. A total of 6 458 patients (44%) received BCS. The proportion of patients treated with BCS was 57%, 37% and 17%, for stage I, II and III cancers, respectively, and was relatively constant over the study period. Regardless of stage, the proportion of patients treated with BCS was highest when surgery occurred in an urban center (Edmonton or Calgary), when treatment with neo-adjuvant chemotherapy was not utilized, and when patients were less than 70 years old.

**Table 1:** **Characteristics of stage I-III breast cancer patients who received breast conserving surgery (BCS)** ^**1**^

	**Stage I**		**Stage II**		**Stage III**	
	**BCS**	**Total**	**BCS**	**Total**	**BCS**	**Total**
	**N (%)** ^**2**^	**N**	**N (%)** ^**2**^	**N**	**N (%)** ^**2**^	**N**
**Overall**	4067 (57)	7091	2060 (37)	5619	331 (17)	1936
**Age at diagnosis**	**P < 0.001**		**P < 0.001**		**P = 0.018**	
<50	880 (58)	1509	656 (40)	1638	112 (18)	628
50-59	1171 (63)	1853	585 (41)	1417	101 (20)	505
60-69	1051 (58)	1801	430 (38)	1144	66 (18)	370
70-79	697 (50)	1394	248 (29)	861	27 (11)	251
≥80	268 (50)	534	141 (25)	559	25 (14)	182
**Geography of surgery**	**P < 0.001**		**P < 0.001**		**P = 0.20**	
Calgary	1637 (62)	2659	901 (42)	2156	139 (19)	739
South	289 (50)	577	145 (30)	490	25 (17)	146
Central	178 (39)	461	112 (24)	473	17 (11)	157
Edmonton	1849 (58)	3180	832 (37)	2258	134 (17)	804
North	114 (53)	214	70 (29)	242	16 (18)	90
**Year of diagnosis**	**P = 0.09**		**P = 0.39**		**P = 0.85**	
2002-2004	1277 (56)	2296	632 (36)	1767	86 (16)	522
2005-2007	1315 (58)	2284	668 (36)	1842	124 (18)	701
2008-2010	1475 (59)	2511	760 (38)	2010	121 (17)	713
**ER/PR status**	**P = 0.04**		**P = 0.22**		**P = 0.15**	
Positive	3407 (58)	5885	1686 (37)	4551	266 (18)	1495
Negative	660 (55)	1206	374 (35)	1068	65 (15)	441
**Neo-adjuvant chemotherapy**	**P < 0.001**		**P < 0.001**		**P < 0.001**	
Not received	4060 (58)	7054	1994 (37)	5336	303 (20)	1523
Received	7 (19)	37	66 (23)	283	28 (7)	413

^1^P values reflect Chi square tests of association for BCS versus mastectomy.

^2^The denominator for each percentage is the number of patients in the adjacent row for the same stage of disease.

Table 2 displays the characteristics of breast cancer patients by geographic region of surgery. Most patients received surgery in one of the two urban centers, Calgary (38%) or Edmonton (43%). Patients differed to some extent in age distribution, clinical disease and treatment by geography of surgery. The percentage of patients under age 60 who received surgery in Southern or Central Alberta was 45-47% whereas patients under age 60 comprised 50-55% of the patients in the other regions. The percentage of patients with ER/PR negative disease ranged from a low of 16% in Calgary to a high of 25% in the North. Patients who received surgery in Northern Alberta, were also more likely to have stage II/III cancers (60%), and to receive neo-adjuvant (8%) and adjuvant chemotherapy (43%), compared to patients from other regions.

**Table 2:** **Characteristics of breast cancer patients by geographic region of surgery**

	**Geography of surgery**				
	**Calgary**	**South**	**Central**	**Edmonton**	**North**
	**N (%)**	**N (%)**	**N (%)**	**N (%)**	**N (%)**
**Total**	5554 (38)	1213 (8)	1091 (7)	6242 (43)	546 (4)
**Surgical treatment**					
Mastectomy	2877 (52)	754 (62)	784 (72)	3427 (55)	346 (63)
BCS	2677 (48)	459 (38)	307 (28)	2815 (45)	200 (37)
With Radiation	2381 (89)	369 (80)	253 (82)	2492 (89)	168 (84)
Without Radiation	296 (11)	90 (20)	54 (18)	323 (11)	32 (16)
**Stage**					
I	2659 (48)	577 (48)	461 (42)	3180 (51)	214 (39)
II	2156 (29)	490 (40)	473 (43)	2258 (36)	242 (44)
III	739 (13)	146 (12)	157 (14)	804 (13)	90 (16)
**Age at diagnosis**					
<50	1597 (29)	244 (20)	256 (23)	1522 (24)	156 (29)
50-59	1471 (26)	298 (25)	265 (24)	1603 (26)	138 (25)
60-69	1170 (21)	284 (23)	247 (23)	1487 (24)	127 (23)
70-79	875 (16)	235 (19)	203 (19)	1103 (18)	90 (16)
≥80	441 (8)	152 (13)	120 (11)	527 (8)	35 (6)
**Year of diagnosis**					
2002-2004	1761 (32)	390 (32)	365 (33)	1900 (30)	169 (31)
2005-2007	1824 (33)	405 (33)	354 (32)	2052 (33)	192 (35)
2008-2010	1969 (35)	418 (34)	372 (34)	2290 (37)	185 (34)
**ER/PR status**					
Positive	4659 (84)	975 (80)	846 (76)	5044 (81)	407 (75)
Negative	895 (16)	238 (20)	245 (22)	1198 (19)	139 (25)
**Neo-adjuvant chemotherapy**					
Not Received	5322 (96)	1187 (98)	1035 (95)	5865 (94)	504 (92)
Received	232 (4)	26 (2)	56 (5)	377 (6)	42 (8)
**Adjuvant chemotherapy**					
Not Received	3460 (62)	825 (68)	657 (60)	3962 (63)	312 (57)
Received	2094 (38)	388 (32)	434 (40)	2280 (37)	234 (43)

The stage-specific adjusted relative risk estimates of receiving BCS are displayed in Table 3. For all stages, receipt of BCS was associated with age less than 70 years at diagnosis and not receiving neo-adjuvant chemotherapy treatment. Substantial variation in the receipt of BCS was found by geographic area of surgery for stage I and II disease. The adjusted relative risk of BCS was highest in Calgary and lowest in Central Alberta for all disease stages. Relative to surgeries performed in Calgary, those performed in Central Alberta were significantly less likely to be BCS for stage I (RR = 0.65; 95% CI: 0.57, 0.72), II (RR = 0.58; 95% CI: 0.49, 0.68), and III (RR = 0.62; 95% CI: 0.37, 0.95) disease, respectively, adjusting for age at diagnosis, year of diagnosis, ER/PR status and receipt of neo-adjuvant chemotherapy. In the most recent time period (2008–2010), among stage I and II patients less than 70 years of age, 1601 (48%) patients received mastectomy, while 1707 (52%) patients received BCS; 578 (46%), 152 (57%), 144 (67%), 650 (45%) and 68 (58%) patients from Calgary, Southern, Central, Edmonton and Northern Alberta received mastectomy, as opposed to BCS. BCS uptake among stage I and II patients increased significantly over the study period in Edmonton (P < 0.05), decreased among stage II patients in Central Alberta (P < 0.05) and remained fairly steady in the other regions and in stage III patients over the study period (Additional file 1: Tables S1–1, S1–2 and S1–3).

**Table 3:** **Adjusted** ^**1**^ **relative risk estimates of receiving BCS rather than mastectomy**

	**Adjusted** ^**1**^ **relative risk estimates (95% confidence intervals)**		
	**Stage I**	**Stage II**	**Stage III**
**Age at diagnosis**	**P < 0.001**	**P < 0.001**	**P < 0.001**
<50	1.00	1.00	1.00
50-59	1.08 (1.02, 1.14)	1.03 (0.95, 1.13)	1.10 (0.86, 1.39)
60-69	1.01 (0.95, 1.07)	0.94 (0.86, 1.03)	0.95 (0.72, 1.24)
70-79	0.87 (0.81, 0.93)	0.71 (0.63, 0.80)	0.53 (0.35, 0.77)
≥80	0.87 (0.79, 0.95)	0.62 (0.53, 0.73)	0.63 (0.41, 0.92)
**Geography of surgery**	**P < 0.001**	**P < 0.001**	**P = 0.30**
Calgary	1.00	1.00	1.00
South	0.83 (0.76, 0.90)	0.74 (0.63, 0.85)	0.92 (0.61, 1.32)
Central	0.65 (0.57, 0.72)	0.58 (0.49, 0.68)	0.62 (0.37, 0.95)
Edmonton	0.96 (0.92, 1.00)	0.91 (0.85, 0.98)	0.95 (0.77, 1.18)
North	0.88 (0.77, 0.99)	0.72 (0.58, 0.88)	0.95 (0.57, 1.45)
**Year of diagnosis**	**P = 0.24**	**P = 0.40**	**P = 0.99**
2002-2004	1.00	1.00	1.00
2005-2007	1.02 (0.97, 1.08)	1.02 (0.94, 1.11)	1.02 (0.80, 1.31)
2008-2010	1.04 (0.99, 1.09)	1.06 (0.97, 1.15)	1.02 (0.79, 1.31)
**ER/PR status**	**P = 0.11**	**P = 0.34**	**P = 0.44**
Positive	1.00	1.00	1.00
Negative	0.96 (0.90, 1.01)	0.96 (0.88, 1.04)	0.91 (0.70, 1.15)
**Neo-adjuvant chemotherapy**	**P < 0.001**	**P < 0.001**	**P < 0.001**
Not Received	1.00	1.00	1.00
Received	0.32 (0.15, 0.57)	0.57 (0.45, 0.70)	0.31 (0.21, 0.45)

^1^Adjusted for all variables in the table.

Table 4 displays the relationship between patient age, clinical, and treatment characteristics and their associations with receipt of radiotherapy in the subgroup of patients treated with BCS. Of the 6 458 patients treated with BCS, 88% subsequently received adjuvant radiotherapy. The proportion of patients that received radiotherapy after BCS was 89%, 86%, and 83% for stage I, II and III breast cancers, respectively. Receipt of radiotherapy was highest among patients with ER/PR negative and ER/PR positive cancers treated with hormone therapy, those who received surgery in an urban area (Edmonton or Calgary), those who received adjuvant chemotherapy and in those younger than 80 years of age, regardless of the cancer stage. Patients aged 80 years or older at diagnosis were at least 40% less likely to receive radiation after BCS compared to patients aged 70–79 years at diagnosis in all disease stages.

**Table 4:** **Characteristics of breast cancer patients who received radiotherapy treatment after breast conserving surgery** ^**1**^

	**Stage I**		**Stage II**		**Stage III**	
	**Post-surgical radiation**	**Received BCS**	**Post-surgical radiation**	**Received BCS**	**Post-surgical radiation**	**Received BCS**
	**N (%)** ^**2**^	**N**	**N (%)** ^**2**^	**N**	**N (%)** ^**2**^	**N**
**Overall**	3607 (89)	4067	1780 (86)	2060	276 (83)	331
**Age at diagnosis**	**P < 0.001**		**P < 0.001**		**P < 0.001**	
<50	821 (93)	880	599 (91)	656	93 (83)	112
50-59	1096 (94)	1171	521 (89)	585	94 (93)	101
60-69	971 (92)	1051	392 (91)	430	57 (86)	66
70-79	601 (86)	697	211 (85)	248	23 (85)	27
≥80	118 (44)	268	57 (40)	141	9 (36)	25
**Geography of surgery**	**P < 0.001**		**P = 0.001**		**P = 0.70**	
Calgary	1485 (91)	1637	783 (87)	901	113 (81)	139
South	236 (82)	289	113 (78)	145	20 (80)	25
Central	149 (84)	178	90 (80)	112	14 (82)	17
Edmonton	1640 (89)	1849	738 (89)	832	114 (85)	134
North	97 (85)	114	56 (80)	70	15 (94)	16
**Year of diagnosis**	**P = 0.06**		**P = 0.01**		**P = 0.10**	
2002 - 2004	1148 (90)	1277	565 (89)	632	78 (91)	86
2005 - 2007	1145 (87)	1315	560 (84)	668	101 (81)	124
2008 - 2010	1314 (89)	1475	655 (86)	760	97 (80)	121
**ER/PR Status & Hormone therapy**	**P < 0.001**		**P < 0.001**		**P < 0.001**	
ER/PR positive & received hormone	2337 (93)	2514	1399 (92)	1516	213 (90)	237
ER/PR positive & no hormone	685 (77)	893	79 (46)	170	7 (24)	29
ER/PR negative	585 (89)	660	302 (81)	374	56 (86)	65
**Neo-adjuvant chemotherapy**	**P = 0.57**		**P = 0.36**		**P = 0.10**	
Not received	3601 (89)	4060	1720 (86)	1994	256 (84)	303
Received	6 (86)	7	60 (91)	66	20 (71)	28
**Adjuvant chemotherapy**	**P < 0.001**		**P < 0.001**		**P < 0.001**	
Not received	2951 (87)	3381	617 (75)	823	52 (55)	95
Received	656 (96)	686	1163 (94)	1237	224 (95)	236

^1^P values reflect Chi square tests of association for BCS versus mastectomy.

^2^The denominator for each percentage is the number of patients who received breast conserving surgery in the adjacent row for the same stage of disease.

Table 5 displays the stage-specific adjusted relative risk estimates of receiving radiotherapy after BCS. Patients aged 80 years or older were 51%, 47% and 36% less likely to be treated with adjuvant radiotherapy for stage I (RR = 0.49; 95% CI: 0.43, 0.56), stage II (RR = 0.53; 95% CI: 0.43, 0.64), and stage III (RR = 0.64; 95% CI: 0.34, 1.05) cancers, respectively, compared to those less than 50 years of age, adjusting for age at diagnosis, year of diagnosis, ER/PR status and hormone therapy, neo-adjuvant chemotherapy and adjuvant chemotherapy. Patients who were ER/PR positive but did not receive hormone therapy were 14%, 41% and 65% less likely to receive radiation after BCS in stage I (RR = 0.86; 95% CI: 0.83, 0.89), II (RR = 0.59; 95% CI: 0.49, 0.68), and III (RR = 0.35; 95% CI: 0.16, 0.60) cancers, respectively, than those who were ER/PR positive and received hormone therapy. Treatment with adjuvant chemotherapy was also associated with receipt of radiotherapy, after adjustment, in stage II (RR = 1.08; 95% CI: 1.05, 1.13) and III (RR = 1.37; 95% CI: 1.14, 1.84) cancers, compared to not being treated with adjuvant chemotherapy. No significant geographical variation of post-BCS radiotherapy receipt was found.

**Table 5:** **Adjusted** ^**1**^ **relative risk estimates of receiving radiotherapy treatment after breast conserving surgery**

	**Adjusted** ^**1**^ **relative risk estimates (95% confidence interval)**		
	**Stage I**	**Stage II**	**Stage III**
**Age at diagnosis**	**P < 0.001**	**P < 0.001**	**P = 0.37**
<50	1.00	1.00	1.00
50-59	1.00 (0.98, 1.02)	0.99 (0.96, 1.02)	1.02 (0.96, 1.10)
60-69	0.99 (0.96, 1.01)	1.02 (0.99, 1.04)	1.02 (0.92, 1.10)
70-79	0.94 (0.91, 0.97)	1.01 (0.96, 1.04)	1.03 (0.90, 1.11)
≥80	0.49 (0.43, 0.56)	0.53 (0.43, 0.64)	0.64 (0.34, 1.05)
**Geography of surgery**	**P = 0.09**	**P = 0.07**	**P = 0.87**
Calgary	1.00	1.00	1.00
South	0.96 (0.91, 1.00)	0.94 (0.86, 1.00)	1.03 (0.89, 1.11)
Central	0.96 (0.91, 1.01)	0.97 (0.90, 1.01)	1.03 (0.86, 1.11)
Edmonton	0.99 (0.98, 1.01)	1.00 (0.98, 1.03)	1.03 (0.97, 1.10)
North	0.95 (0.88, 1.00)	0.92 (0.81, 1.01)	1.03 (0.82, 1.12)
**Year of diagnosis**	**P = 0.25**	**P = 0.15**	**P = 0.96**
2002 - 2004	1.00	1.00	1.00
2005 - 2007	0.99 (0.97, 1.01)	0.97 (0.94, 1.00)	1.00 (0.94, 1.06)
2008 - 2010	1.00 (0.98, 1.02)	0.98 (0.96, 1.01)	0.99 (0.92, 1.06)
**ER/PR Status & Hormone therapy**	**P < 0.001**	**P < 0.001**	**P < 0.001**
ER/PR positive & received hormone	1.00	1.00	1.00
ER/PR positive & no hormone	0.86 (0.83, 0.89)	0.59 (0.49, 0.68)	0.35 (0.16, 0.60)
ER/PR negative	0.94 (0.91, 0.97)	0.89 (0.84, 0.93)	0.95 (0.85, 1.02)
**Neo-adjuvant chemotherapy**	**P = 0.33**	**P = 0.04**	**P = 0.86**
Not received	1.00	1.00	1.00
Received	0.89 (0.52, 1.05)	1.10 (1.00, 1.14)	1.03 (0.72, 1.47)
**Adjuvant chemotherapy**	**P = 0.62**	**P < 0.001**	**P < 0.001**
Not received	1.00	1.00	1.00
Received	1.01 (0.98, 1.02)	1.08 (1.05, 1.13)	1.37 (1.14, 1.84)

^1^Adjusted for all variables in the table.

## Discussion

In this study, 44% of early stage breast cancer patients diagnosed from 2002 to 2010 in Alberta received breast conserving surgery, as opposed to mastectomy (57%, 37% and 17% of stage I, II and III patients, respectively). Similar population-based results have been reported in the Netherlands, where 48% of patients diagnosed between 2003 and 2006 received BCS (63%, 41% and 19% of stage I, II and III patients, respectively) [24]. BCS receipt among patients in the United States is higher, with estimates ranging from 50% to 70% [17,25-28]. Within Canada, estimates range from 74% in Quebec to 31% and 35% in Newfoundland and Saskatchewan, respectively [29]. Tyldesley *et al.* (2003) have estimated that in North America, 70% of all breast cancers are eligible for BCS, and 48 ± 6% of cases are both eligible and prefer it to mastectomy [30]. The great variation present within and between countries, however, suggests that there is not a consensus on standard practice.

In a publicly-funded health care system, factors such as region of residence should not be related to the receipt of standard treatment. This study found significant variation in surgical breast cancer treatment received by geography in Alberta, while finding no apparent geographical variation in the receipt of post-BCS radiotherapy. The consistent finding across disease stage that the highest rate of mastectomy is in Central Alberta is rather surprising. Poor access to radiation therapy has been associated with low BCS rates in other studies [25,27,28], and may partially explain the geographic variation we found. At the time of this study, however, patients in Central Alberta did not have worse access to radiation facilities compared to those in Northern or Southern Alberta; in fact, many patients in Central Alberta live significantly closer to radiation facilities than those in Southern or Northern Alberta, suggesting better access. While poor access to radiation therapy may partially explain the lower BCS rates in Northern and Southern Alberta which have lower population densities than Central Alberta, the lower rates in Central Alberta suggests that distance to radiotherapy is not the only important factor influencing choice of surgical treatment.

Geographic variation in the receipt of breast conserving surgery has also been found in other health care systems, including in the Netherlands [24] and in Switzerland [31]. The results from both of these studies also suggest that distance to radiotherapy is not the primary reason for the observed geographic variation; the authors suggest instead that provider-related factors are a more likely explanation. We make the same hypothesis for the present results in Alberta, factors such as provider volume and preferences, which have been associated with patient surgery type received, influence the receipt of BCS by breast cancer patients differentially across the province [32,33]. Publicly-funded health care systems do not ensure equity of care by region, and effort is needed to further investigate and address these inequities. Since 2010, Alberta Health Services has increased the number of sites which provide radiotherapy across the province. Investigation into how this affects geographical variation of BCS receipt in the future will help elucidate the impact of access to radiation compared to other factors.

Of patients who received BCS, 88% subsequently received radiation therapy. In comparison, it can be estimated that around 80% of patients from the United States receive post-BCS radiation [15,17,27], and it has been reported that 99% of patients in the Netherlands received this treatment in 2008, rising from 87% in 1997 [34]. These estimates suggest that physicians collectively recognize the importance of radiotherapy treatment after BCS. We found that the proportion of patients who received post-BCS radiotherapy dropped dramatically with age greater than 80 years, however. This can be attributed to unclear advantages of radiotherapy in this population when life expectancy, potential toxicity and comorbidities are taken in to account. There has been substantial recent discussion as to whether or not postoperative radiotherapy can be reasonably omitted in some women over the age of 70 with low-risk breast cancer [35-37].

A great strength of this study is that it is population-based and thus contains the entire population of interest in Alberta over an eight year period. In practice, treatment choices are made based on various factors including patient preference, feasibility (e.g. distance and access to transportation), and the presence of comorbidities. It is, therefore, of clinical interest to investigate treatments received in a population based series of patients. In addition to this, the data source used is known for being very complete and accurate, as the Alberta Cancer Registry captures 99.9% of cancer cases. A limitation of the study, however, was our inability to assess patient preferences and contraindications for breast-conserving surgery due to the nature of the data source. For example, there may be residual confounding of the BCS geography association by tumor size beyond that explained by stage as patients with very large tumors may be more likely to reside in rural areas. We were also not able to account for confounding by economic status, race or surgeon preference, all of which may explain some of the geographic variation seen. Further interpretation of the BCS receipt results could have been achieved if a subgroup analysis had been performed, in which patients with contraindications for BCS, such as those with very large tumors, were excluded.

## Conclusions

This study found significant geographic variation in BCS receipt and consistently high rates of adjuvant radiation in those who received BCS across the province. Overall, treatment patterns did not change between 2002 and 2010, although some area-specific trends were found. Further investigation is needed to understand the low rates of breast-conserving surgery in certain parts of the province, such as Central Alberta, compared to other parts in order to identify appropriate interventions. This study provides strong baseline information on variation in rates of BCS and subsequent radiation therapy in Alberta over an eight year time period which will be useful for evaluating the effectiveness of interventions that are implemented in the future.

## Additional file

Additional file 1: Table S1-1. Adjusted^1^ relative risk estimates of receiving BCS rather than mastectomy by geography of surgery, among stage I patients. **Table S1-2.** Adjusted^1^ relative risk estimates of receiving BCS rather than mastectomy by geography of surgery, among stage II patients. **Table S1-3.** Adjusted^1^ relative risk estimates of receiving BCS rather than mastectomy by geography of surgery, among stage III patients.

## Abbreviations

BCS: Breast-conserving surgery
CI: Confidence interval
ER: Estrogen receptor
PR: Progesterone receptor
RR: Relative risk
