# Supplementary material for: Treatment variation in patients diagnosed with early stage breast cancer in Alberta from 2002 to 2010: a population-based study
Source: BMC Health Serv Res. 2015 Jan 22;15:35. doi: 10.1186/s12913-015-0680-z (PMC4308832; doi:10.1186/s12913-015-0680-z)
Supplement: Additional file 1: Table S1-1. — Adjusted1 relative risk estimates of receiving BCS rather than mastectomy by geography of surgery, among stage I patients. Table S1-2. Adjusted1 relative risk estimates of receiving BCS rather than mastectomy by geography of surgery, among stage II patients. Table S1-3. Adjusted1 relative risk estimates of receiving BCS rather than mastectomy by geography of surgery, among stage III patients. [file 12913_2015_680_MOESM1_ESM.docx]

**Additional file**

**Table S1-1.** Adjusted^1^ relative risk estimates of receiving BCS rather than mastectomy by geography of surgery, among stage I patients

|  | **Adjusted^1^ Relative Risk Estimates**  **(95% Confidence Intervals)** | | |
| --- | --- | --- | --- |
|  | **2002-2004** | **2005-2007** | **2008-2010** |
| **Geography of Surgery** | **P < 0.001** | **P < 0.001** | **P < 0.001** |
| Calgary | 1.00 | 1.00 | 1.00 |
| South | 0.73 (0.61 , 0.85) | 1.00 (0.87 , 1.14) | 0.76 (0.65 , 0.88) |
| Central | 0.72 (0.59 , 0.85) | 0.59 (0.46 , 0.74) | 0.60 (0.48 , 0.73) |
| Edmonton | 0.89 (0.82 , 0.96) | 0.99 (0.92 , 1.07) | 0.99 (0.93 , 1.06) |
| North | 0.76 (0.58 , 0.96) | 0.99 (0.79 , 1.18) | 0.87 (0.68 , 1.06) |

1. Adjusted for age at diagnosis, ER/PR status and adjuvant chemotherapy

**Table S1-2.** Adjusted^1^ relative risk estimates of receiving BCS rather than mastectomy by geography of surgery, among stage II patients

|  | **Adjusted^1^ Relative Risk Estimates**  **(95% Confidence Intervals)** | | |
| --- | --- | --- | --- |
|  | **2002-2004** | **2005-2007** | **2008-2010** |
| **Geography of Surgery** | **P < 0.001** | **P < 0.001** | **P < 0.001** |
| Calgary | 1.00 | 1.00 | 1.00 |
| South | 0.72 (0.55 , 0.92) | 0.81 (0.62 , 1.02) | 0.68 (0.52 , 0.87) |
| Central | 0.76 (0.59 , 0.96) | 0.50 (0.35 , 0.68) | 0.49 (0.35 , 0.65) |
| Edmonton | 0.77 (0.67 , 0.88) | 1.04 (0.92 , 1.18) | 0.92 (0.82 , 1.03) |
| North | 0.74 (0.51 , 1.02) | 0.84 (0.59 , 1.13) | 0.60 (0.39 , 0.85) |

1. Adjusted for age at diagnosis, ER/PR status and adjuvant chemotherapy

**Table S1-3.** Adjusted^1^ relative risk estimates of receiving BCS rather than mastectomy by geography of surgery, among stage III patients

|  | **Adjusted^1^ Relative Risk Estimates**  **(95% Confidence Intervals)** | | |
| --- | --- | --- | --- |
|  | **2002-2004** | **2005-2007** | **2008-2010** |
| **Geography of Surgery** | **P = 0.87** | **P = 0.22** | **P = 0.10** |
| Calgary | 1.00 | 1.00 | 1.00 |
| South | 1.08 (0.52 , 1.97) | 0.89 (0.43 , 1.60) | 0.80 (0.35 , 1.50) |
| Central | 0.85 (0.39 , 1.61) | 0.57 (0.21 , 1.20) | 0.41 (0.13 , 0.95) |
| Edmonton | 0.88 (0.56 , 1.36) | 1.19 (0.85 , 1.67) | 0.79 (0.55 , 1.12) |
| North | 0.63 (0.16 , 1.59) | 0.60 (0.15 , 1.48) | 1.40 (0.74 , 2.33) |

1. Adjusted for age at diagnosis, ER/PR status and adjuvant chemotherapy
